# Prenatal Bisphenol A Exposure is Linked to Epigenetic Changes in Glutamate Receptor Subunit Gene *Grin2b* in Female Rats and Humans

**DOI:** 10.1038/s41598-018-29732-9

**Published:** 2018-07-27

**Authors:** Ali Alavian-Ghavanini, Ping-I Lin, P. Monica Lind, Sabina Risén Rimfors, Margareta Halin Lejonklou, Linda Dunder, Mandy Tang, Christian Lindh, Carl-Gustaf Bornehag, Joëlle Rüegg

**Affiliations:** 1 Swetox, Karolinska Institutet, Unit of Toxicology Sciences, Forskargatan 20, 151 36 Södertälje, Sweden; 2 Karolinska Institutet, Department of Clinical Neuroscience, Centre for Molecular Medicine (CMM), 171 64 Solna, Sweden; 3 Karlstad University, Department of Health Sciences, 651 88 Karlstad, Sweden; 4 Uppsala University, Department of Medical Sciences, Occupational and Environmental Medicine, 751 85 Uppsala, Sweden; 5 Lund University, Division of Occupational and Environmental Medicine, Lund University, 221 85 Lund, Sweden; 6 Icahn School of Medicine at Mount Sinai, New York, NY USA

## Abstract

Bisphenol A (BPA) exposure has been linked to neurodevelopmental disorders and to effects on epigenetic regulation, such as DNA methylation, at genes involved in brain function. High doses of BPA have been shown to change expression and regulation of one such gene, *Grin2b*, in mice. Yet, if such changes occur at relevant doses in animals and humans has not been addressed. We investigated if low-dose developmental BPA exposure affects DNA methylation and expression of *Grin2b* in brains of adult rats. Furthermore, we assessed associations between prenatal BPA exposure and *Grin2b* methylation in 7-year old children. We found that *Grin2b* mRNA expression was increased and DNA methylation decreased in female, but not in male rats. In humans, prenatal BPA exposure was associated with increased methylation levels in girls. Additionally, low APGAR scores, a predictor for increased risk for neurodevelopmental diseases, were associated with higher *Grin2b* methylation levels in girls. Thus, we could link developmental BPA exposure and low APGAR scores to changes in the epigenetic regulation of *Grin2b*, a gene important for neuronal function, in a sexual dimorphic fashion. Discrepancies in exact locations and directions of the DNA methylation change might reflect differences between species, analysed tissues, exposure level and/or timing.

## Introduction

Bisphenol A (BPA) is a widely used chemical in the production of polycarbonate plastics and epoxy resins existing in many common consumers and household products^1^. Consequently, humans are exposed to low levels of BPA on a daily basis. For instance, a population-based study in the U.S. showed that over 92% of children over the age of 6 had detectable levels of BPA in their urine^2^. Estimated BPA exposure levels are 0.01–0.4 µg/kg body weight (bw)/day for adults and 0.1–0.5 µg/kg bw/day for children and adolescents^3^.

Numerous studies have demonstrated that BPA interferes with estrogen signalling and other hormonal signalling pathways, both *in vitro* and in animals, thus acting as an endocrine disrupting chemical (EDC)^4–6^. BPA targets, such as the estrogen system, are crucially involved in neurodevelopment, in particular in sexual differentiation of the brain^7,8^. Thus, interference with these systems during sensitive developmental periods could ultimately affect brain function and behaviour. Indeed, a number of epidemiological and experimental studies suggest that early life exposure to BPA might lead to behavioural alteration later in life^6,9–12^. In the majority of studies, the effects of BPA were sex-specific, often blunting or abolishing natural sexual dimorphisms^12,13^.

It is also known that BPA induces epigenetic changes, which are thought to underlie persistent effects of early life exposures^13–16^. Epigenetic processes drive cell differentiation by regulating long-term gene function, and are crucial for foetal development, including the brain^17,18^. Epigenetic marks regulate gene transcription in different ways. E.g., DNA methylation at transcription factor (TF) binding sites in regulatory regions can prevent TF binding and thus decrease gene transcription. There is ample evidence that BPA induces epigenetic changes in a variety of experimental systems, and several epidemiological studies indicate a link between epigenetic changes, in particular DNA methylation alterations, and chemical exposures including BPA^16,19^. In animal studies, BPA has been shown to affect DNA methylation of genes involved in brain development and function, such as *Bdnf*, *Fkbp5*, and *Grin2b*^20–22^.

*Grin2b* is a particularly interesting target as genetic polymorphisms in this gene have been consistently found to be associated with neurodevelopmental diseases/disorders, such as Attention Deficit Hyperactivity Disorder (ADHD), Autism Spectrum Disorder (ASD), and schizophrenia^23–25^. The *Grin2b* gene encodes the NR2B subunit of N-methyl-D-aspartate receptors (NMDARs), which are receptors for the excitatory neurotransmitter glutamate and important for regulation of neural morphology, learning and memory^26^. Its transcription is, among others, regulated by the TF nuclear respiratory factor 1 (Nrf1)^27^, whose DNA binding, in turn, is inhibited by DNA methylation^28^.

Glutamate receptor genetic functional variations may hold the key to glutamate status^24^, which could underlie their association with psychiatric disorders. For example, higher glutamate concentrations have been found in patients with schizophrenia^29,30^. Intriguingly, perinatal exposure to BPA has also been suggested to cause an increase in glutamate concentration in the rat hippocampus^31^. However, it remains unclear if changes in NMDAR composition contributes to this increase, and if epigenetic dysregulation of their subunit genes is involved. Furthermore, a link between BPA exposure and *Grin2b* expression or methylation has, to our knowledge, never been addressed in humans.

Here, we assessed whether developmental exposure to BPA in doses around or below the human tolerable daily intake set by the European Food Safety Authority (EFSA) (4 μg/kg bw/day) and the U.S. Food and Drug Administration (FDA) (50 μg/kg bw/day) persistently changes expression and methylation of *Grin2b* in Fischer 344 rat brain. Furthermore, we explored if prenatal BPA exposure is associated with DNA methylation of *GRIN2B* in 7-year old children, when adjusting for potential confounders.

## Results

### Effects of BPA on *Grin2b* in rats

**Figure 1:**
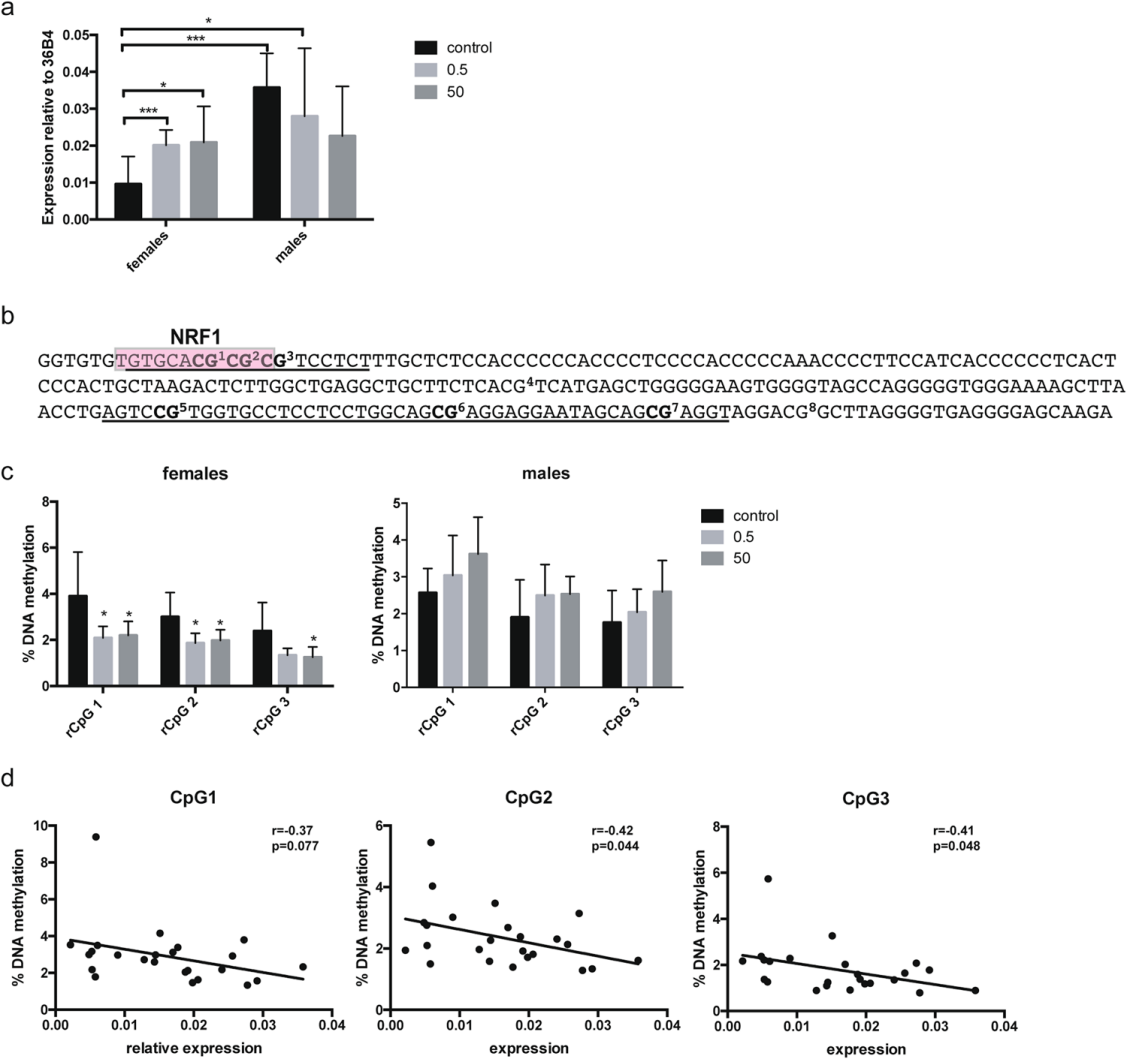
Developmental BPA exposure leads to changes in Grin2b expression and methylation in hippocampus of female rats one year after exposure had ceased. (**a**) Gene expression analysis of *Grin2b* assessed by qPCR in hippocampus of 52 weeks old rats developmentally exposed to 0.5 or 50 μg/kg bw/day BPA or vehicle control. Bars show mean and standard deviation of *Grin2b* expression relative to the control gene 36B4 for female (control: n = 10, 0.5: n = 7, 50: n = 8) and male (control: n = 12, 0.5: n = 8, 50: n = 7) animals. (**b**) Sequence of the two adjacent regions (underlined) analysed for DNA methylation in the rat *Grin2b* promoter. The predicted Nrf1 binding site is marked with a red box. (**c**) DNA methylation analysis of 3 CpGs in the promoter region of *Grin2b* assessed by bisulfite pyrosequencing in hippocampus of 52 weeks old rats developmentally exposed to 0.5 or 50 μg/kg bw/day BPA or vehicle control. Bars show mean and standard deviation of % methylation for female (control: n = 12, 0.5: n = 8, 50: n = 8) and male (control: n = 8, 0.5: n = 8, 50: n = 8) animals. (**d**) Correlation between relative *Grin2b* expression and methylation at CpG 1-3 in female rat hippocampus. *p < 0.05, **p < 0.01, ***p < 0.001 treatment animals compared to controls.

In order to identify persistent changes in *Grin2b* upon developmental BPA treatment, DNA methylation and gene expression were analysed in 52 weeks old Fischer 344 rats. We used hippocampus for these analyses as this is a region expressing *Grin2b* even in the adult brain^32^. Dams were exposed to 0.5 or 50 μg BPA/kg bw/day or vehicle (control) during gestation and lactation via their drinking water. No differences in body weight and other gross health parameters were observed between the treatment groups (see Supplementary Table S1). Analysis of hippocampal *Grin2b* expression showed that both doses of BPA lead to a significant increase in *Grin2b* expression in females (p = 0.0094 control vs. 0.5 μg and p = 0.0033 control vs. 50 μg) whereas no significant change in males was detected, although a trend towards decreased expression was observed (Fig. 1a). This opposing effect of BPA in females vs. males led to elimination of a significant sex difference in *Grin2b* expression (p < 0.0001) observed in the control animals.

To explore the role of epigenetic regulation for this change in *Grin2b* expression, we analysed DNA methylation at two adjacent regions in the *Grin2b* promoter, one containing a predicted Nrf1 binding site and one that is homologous to a region in mice that has previously been shown to be affected by BPA (depicted in Fig. 1b)^22^. DNA methylation analysis, performed in two independent runs, showed that prenatal BPA exposure at both doses leads to a significant decrease in methylation in females in the three CpGs that are part of the Nrf1 binding site (CpG1: p = 0.019 control vs. 0.5 μg and p = 0.036 control vs. 50 μg; CpG2: p = 0.011 control vs. 0.5 μg and p = 0.03 control vs. 50 μg; CpG3: p = 0.053 control vs. 0.5 μg and p = 0.035 control vs. 50 μg). No significant changes were observed in males (Fig. 1c). For the CpGs in the adjacent region (rCpGs 5–7, homologous to the ones analysed in^22^), no significant methylation differences were found in neither of the sexes (see Supplementary Fig. S1). These results did not change when normalised for potential batch effects using rank-based inverse transformation of the methylation data. *Grin2b* expression and methylation at CpG1-3 in females was inversely correlated (CpG1: p = 0.077; CpG2: p = 0.044; CpG3: p = 0.048) (Fig. 1d), indicating that methylation at these CpGs is functionally involved in gene expression. No significant correlation was found between *Grin2b* expression and methylation at all analysed CpGs in males (see Supplementary Fig. S1).

Developmental exposure to BPA has been shown to affect growth^33^. To exclude that the observed effects are a result of growth differences between the treatment groups, *Grin2b* expression and methylation was adjusted to the animals’ growth rates. Yet, the increase in *Grin2b* expression and decrease in DNA methylation in female rats in the treatment groups remained significant (see Supplementary Fig. S1).

Taken together, our data suggest that developmental exposure to BPA changes the DNA methylation level in the *Grin2b* promoter, resulting in altered gene expression levels in female, but not in male, rats one year after the exposure had ceased.

### Prenatal BPA levels and *GRIN2B* methylation in children

Encouraged by the findings from the rat model, we attempted to assess if prenatal BPA exposure levels were associated with methylation level of the *GRIN2B* gene also in humans, in a subpopulation of the SELMA study^34^ (Table 1). Creatinine adjusted BPA concentrations were measured in the 1^st^ trimester urine in pregnant mothers. Based on these measurements, the daily intake of BPA for mothers of boys and girls were estimated to be 0.007–0.2 µg/kg/day and 0.004–0.6 µg/kg/day, respectively (no significant difference between the two groups of mothers was noticed). Thus, the highest intake measured in the human cohort corresponded to the lower dose given in the rat study. *GRIN2B* methylation was assessed at 3 CpG sites in a region homologous to the investigated rat locus (Fig. 1b), using buccal DNA from 7 year-old children. We identified 4 DNA methylation clusters since the CH pseudo-F value abruptly increased at this number of clusters for all CpG sites (see Supplementary Fig. S3). The methylation levels in each cluster are presented in Supplementary Table S2. Subsequent analyses were conducted separately for females and males to evaluate sex-specific differences.

**Table 1:** Characteristics of 317 children from the SELMA pregnancy cohort.

Sample Characteristics	Females	Males
Number of subjects N (%)	152 (47.8)	165 (51.9)
CpG1 (%) Mean (SD)	2.83 (3.96)	3.54 (4.26)
CpG2 (%) Mean (SD)	2.69 (4.48)	3.09 (4.29)
CpG2 (%) Mean (SD)	1.13 (2.63)	0.87 (2.09)
Bisphenol A (ng/mL) Geometric mean (95% CI)	1.67 (1.47–1.89)	1.62 (1.41–1.87)
Week of enrolment Mean (SD)	9.95 (2.11)	10.2 (2.45)
**5** **minutes APGAR score N (%)**		
*Healthy (score* = *10)*	134 (88.2)	145 (87.9)
*Complication (score* < *10)*	18 (11.8)	20 (12.1)
**Maternal smoking status during pregnancy N (%)**		
*No*	127 (83.6)	138 (83.6)
*Passive*	12 (7.9)	12 (7.3)
*Active*	8 (5.3)	11 (6.7)
**Stressful events during pregnancy N (%)**		
*No*	128 (84.2)	148 (89.7)
*Yes*	24 (15.8)	17 (10.3)

BPA values were adjusted for creatinine.

**Figure 2:**
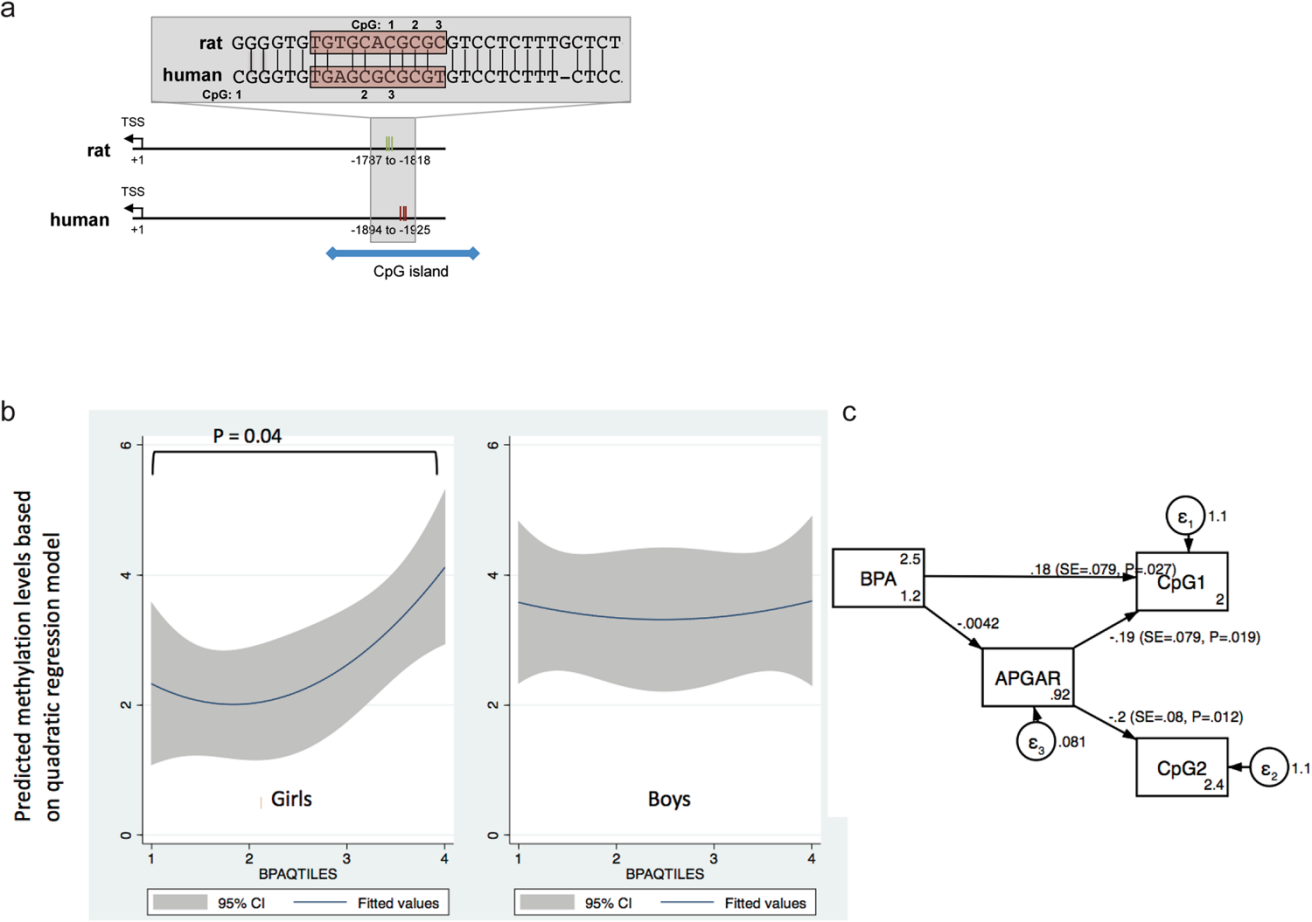
*Relationship between GRIN2B methylation patterns and prenatal BPA levels*. (**a**) Genomic location and sequence of the region analysed for DNA methylation relative to the transcriptional start site (TSS) of rat and human *GRIN2B*. The predicted Nrf1 binding site is marked with a red box. Despite lying in a well-conserved region, only rat (r) CpG 1 (corresponding to human (h) CpG 3) is conserved between the two species. In human, the analysed region is part of a CpG island. (**b**) Sex-dependent correlations between methylation cluster at the first CpG site, using the quadratic regression model. (**c**) Structure equation model (SEM) showing how prenatal BPA levels and APGAR scores at the 5^th^ minute could jointly influence methylations at two CpG sites simultaneously.

Ordered logistic regression analysis results showed a significant association (p = 0.049 and p = 0.039 treating BPA quartile as an ordered categorical variable and dummy variable, respectively) between the methylation level of the first CpG site (denoted as hCpG1) and prenatal BPA levels (expressed both in a continuous scale as well as in quartiles and adjusted for creatinine) in female subjects, after adjusting for potential confounders (Table 2). As shown in Fig. 2a, BPA levels were positively correlated with hCpG1 methylations levels in girls but not in boys.

**Table 2:** Association between prenatal BPA exposure and GRIN2B methylation, adjusted for potential confounders in each ordered logistic regression, expressed as OR (95% CI).

		All subjects	Females only	Males only
Model 1	Predictors	OR (95% CI)	OR (95% CI)	OR (95% CI)
	BPA^φ^ (quartile was treated as an ordered categorical variable)	1.11 (0.91–1.35)	**1**.**36 (1**.**02–1**.91)*	0.93 (0.71–1.20)
	APGAR score (5th minute; 10 vs. <10)	0.61 (0.30–1.25)	**0**.**27 (0**.**09–0**.80)*	1.52 (0.56–4.11)
	Maternal smoking	0.68 (0.44–1.06)	0.79 (0.40–1.56)	0.55 (0.30–1.02)
	Maternal stress	1.20 (0.65–2.22)	0.75 (0.31–1.82)	2.39 (0.95–6.03)
Model 2	BPA quartile (dummy variable)			
	2nd quartile vs. 1st quartile	0.93 (0.50–1.74)	0.95 (0.35–2.52)	0.78 (0.33–1.83)
	3rd quartile vs. 1st quartile	0.73 (0.39–1.37)	0.78 (0.28–2.15)	0.59 (0.25–1.36)
	4th quartile vs. 1st quartile	1.50 (0.82–2.76)	**2**.**72 (1**.**05–7**.01)*	0.85 (0.37–1.93)
	APGAR score (5th minute; 10 vs. < 10)	0.56 (0.27–1.15)	**0**.**21 (0**.**07–0**.65)**	1.49 (0.55–4.06)
	Maternal smoking	0.68 (0.43–1.07)	0.84 (0.42–1.67)	**0**.**53 (0**.**29–0**.99)*
	Maternal stress	1.23 (0.66–2.29)	0.78 (0.32–1.92)	2.40 (0.92–6.10)

*p-value < 0.05, **p-value < 0.01.

^φ^BPA values were adjusted for creatinine before they were assigned into a particular quartile.

### Prenatal BPA levels, APGAR scores, and *GRIN2B* methylation

We also noted that methylation levels at hCpG2 were significantly inversely correlated with APGAR scores, an index for infant’s cardiovascular and pulmonary functions outside the womb (odds ratio = 0.21, p = 0.007) (Table 2). To assess the relationship between BPA and APGAR scores to predict *GRIN2B* methylation at hCpG1 and hCpG2, we used SEM, selecting methylation levels at these two sites as the two primary outcomes, while prenatal BPA levels and APGAR scores were treated as two primary predictors. We also evaluated if APGAR scores could serve as a mediator for the BPA effect on *GRIN2B* methylation. APGAR scores were significantly associated with the methylation levels at both CpG sites (p = 0.019 for hCpG1 and p = 0.012 for hCpG2), whereas prenatal BPA levels were only significantly associated with methylation levels at hCpG1 (p = 0.027) (Fig. 2b), implying that APGAR score and prenatal BPA exposure act as two independent predictors for the methylation at hCpG1 of *GRIN2B* in girls.

## Discussion

In the current study, we assessed effects of early life BPA exposure on methylation levels of the *Grin2b* gene in brain tissue from rats and buccal samples from humans. In the rat hippocampus, we observed that developmental BPA exposure lead to significantly decreased methylation levels in *Grin2b* in females, but not in males. Furthermore, decreased methylation in female rats was concomitant with increased mRNA expression levels. These findings and the observed inverse correlation between *Grin2b* mRNA expression and methylation at rCpG1-3 is a strong indication that methylation at this region is important for transcriptional regulation and responsive to early life BPA exposures in females. Notably, rCpGs 1-3 are part of a predicted binding site for Nrf1, which has been shown to regulate *Grin2b* expression^27^ and to be sensitive to DNA methylation^28,35,36^. Thus it is possible that the correlation between methylation at these CpGs and *Grin2b* mRNA expression in females is due to diminished Nrf1 binding. Nrf1 is not the only TF predicted to bind to this region, but the only one with a documented link to *Grin2b* and brain development and a binding site that is conserved between rat and human (see Supplementary Table S3).

In the human samples, we investigated DNA methylation at a homologous region in the *GRIN2B* gene. The analysed CpGs lie in a CpG island and in the only region nearby the *GRIN2B* gene that is predicted to be a strong promoter, based on data sets relevant for transcriptional regulation produced in the ENCODE project^37^ (see Supplementary Fig. S2). In the human data, we also observed a sexual dimorphic link where prenatal BPA exposure was associated to *GRIN2B* methylation in 7-year old girls, but not in boys of the same age. However, in humans, higher prenatal BPA exposure was associated with higher methylation levels.

The fact that we found opposite associations between BPA exposure and *Grin2b* methylation levels in humans compared to rats could be due to several reasons. Firstly, this could reflect differences between species, similarly to what was found in a previous study comparing DNA methylation of repetitive elements between humans and mice^38^. Of note, only rat (r)CpG1 corresponding to human (h) CpG3, is conserved between humans and rats. Human CpG1 (where associations to BPA levels were found) is not conserved between primates and rodents (Fig. 1b). Thus, the methylation changes, despite affecting a homologous region, were not found at the exact same CpGs. Secondly, the DNA methylation analyses were not conducted at the same age in human subjects and in the animals. While the rats were sacrificed at adulthood (52 weeks of age), the human samples were taken from 7-year old children. It is known that the NMDA receptor subunit 2b, which is encoded by *Grin2b*, is predominantly expressed during late prenatal and early postnatal development and replaced by another subunit later on in life, which changes the signalling properties of the receptor^26,39^. Therefore, the observed increase in *Grin2b* expression in the adult rats could disturb the composition of the NMDA receptors and lead to an altered signalling function. If a similar correlation between methylation and expression of *Grin2b* exists in humans, we might speculate that BPA exposure leads to decreased *Grin2b* levels during early life where the protein encoded by this gene has important functions. Thirdly, even though we are focusing on prenatal exposure for BPA as a single chemical in the human data, it should be kept in mind that real life exposure is a matter of complicated mixtures. For example, we have shown that several alkyl phenols (including BPA) and more than 10 phthalate metabolites were all identified in prenatal urine of more than 2,300 pregnant women enrolled in the SELMA study during the period 2007–2010^40^. In addition, exposures to these compounds are often correlated^41^. In contrast, in experimental animal experiments, other exposures can be largely excluded and controlled for. Thus, while in the animals changes in *Grin2b* methylation can be truly assigned to BPA exposure only, the associations observed in humans are most likely due to mixed exposures.

Furthermore, the DNA methylation analyses were performed in different tissues, and there is limited data on the correlations between brain and buccal DNA methylation patterns. Nevertheless, a number of studies have addressed the relationship between DNA methylation patterns in blood and different brain areas, and there are public resources, such as the Blood Brain DNA Methylation Comparison Tool (ref.^42^ based on the data by Hannon *et al*.^43^) where correlations between these two tissues can be interrogated. Using this tool we found that hCpG1 methylation is significantly correlated between blood and 2 out of 4 assessed brain areas: prefrontal cortex and superior temporal gyrus (see Supplementary Fig. S2), based on around 70 individuals. Further, employing data from other published sources^44,45^, we found a significant positive correlation between blood and saliva methylation at hCpG1 (based on 17 individuals) (see Supplementary Fig. S2). No correlation was found for blood/saliva and buccal epithelial, however, we could only identify one study based on 5 individuals addressing this relationship^45^. Taken together, the available data suggest that the methylation at hCpG1 correlates between different tissues, implicating that the increased methylation at hCpG1 found in buccal epithelial of higher exposed girls could reflects an increase at this site in the target tissue (brain).

Our finding of sex-dependent effects of BPA on the DNA methylation pattern of *Grin2b*, observed in both human and animal data, are in line with previous studies in humans showing sexual-dimorphic effects of BPA on both DNA methylation^9^ and neurodevelopmental outcomes^10^. Human data is supported by findings in rodents, suggesting that persistent changes in brain functions by early life BPA exposure are sex-specific, often blunting or abolishing natural sexual dimorphisms^12,31,46,47^. Together, these findings emphasize the importance of investigating the impact of BPA and other EDCs on the two sexes separately.

We also found that the APGAR score was associated with methylation levels at hCpG1 and hCpG2 within the *GRIN2B* gene. Low APGAR scores might reflect hypoxic events occurring in the uterus or the process of delivery, and hypoxia has been found to lead to global hypermethylation in some tissues^48–50^. Furthermore, in a genome-wide methylation analysis, methylation levels at specific sites were directly associated with the length of birth, i.e. duration of hypoxic condition^51^. Most prior clinical epigenetic studies have treated APGAR scores as a covariate. However, APGAR scores at the fifth minute have been found to predict long-term neurological disabilities and cognitive functions^52^ and the risk for neurodevelopmental disorders, such as ADHD^53^ and ASD^54^. Interestingly, several studies have reported associations between genetic variants in the *GRIN2B* gene and increased risks for neurodevelopmental disorders^24^. It remains to be determined if methylation of *GRIN2B* is also a predictor for such risks or if it is even underlying associations between hypoxic events or BPA exposure and neurodevelopmental impairments.

### Limitations

One limitation of this study is the lack of behavioural assays to evaluate the BPA effect on the phenotype in the same rats as the changes in *Grin2b* methylation and expression were measured. Nevertheless, the literature suggests that BPA exposure in similar experimental settings leads to changes in behaviour and brain function^12,31,46,47^.

Additionally the study does not address molecular events leading to the observed DNA methylation changes upon BPA exposure. Published results show that BPA can affect enzymes involved in the regulation of DNA methylation in rodent brain, such as the level of Dnmt1^55^ and localization of Tet2^56^. Additionally, we have shown that BPA affects DNA methylation of specific genes via ERβ^21,57^, by recruitment of thymine DNA glycosylase, an enzyme involved in DNA demethylation, to specific genomic loci^57^. These are all possible mechanisms underlying the findings presented here. Potentially, effects on *Grin2b* expression and regulation could also be results of other organismal changes, such as metabolic alterations, induced by the exposure rather than direct actions of BPA on DNA methylation regulation. Anyhow, by adjusting for growth parameters, we could exclude their involvement on the observed differences on *Grin2b*.

Furthermore, the inaccessibility of brain tissues in humans forced us to measure DNA methylation in a peripheral tissue (i.e., buccal epithelium). However, our analyses using published data (see above) suggest that methylation at hCpG1 in the *GRIN2B* gene is correlated across different tissues.

Finally, this study has not addressed the functional implications of the DNA methylation changes in humans, neither its effect on gene expression nor its association to neurodevelopmental health outcomes. Nevertheless, the current findings provide a basis for further research on the link between prenatal BPA exposure, DNA methylation patterns in the *GRIN2B* gene, and neurodevelopment outcomes.

## Conclusion

By combining an animal model and human based epidemiologic data we show here that developmental BPA exposure may influence methylation patterns at a regulatory region of the *Grin2b* gene. In both rats and humans, the effect of early life BPA exposure on *Grin2b* methylation was only seen in females, however, there were species-specific differences in location and direction of the methylation changes. In humans, *GRIN2B* methylation was also associated with a low APGAR score, which has been documented as a risk factor for neurodevelopmental diseases. Future research is warranted to clarify if the changes in *GRIN2B* methylation reported in the present study can be linked to impaired neurodevelopment or behavioural deficits associated with prenatal BPA exposure or poorer APGAR scores.

## Material and Methods

### Animal treatment

Animal housing and treatment are described elsewhere^58,59^. Briefly, female F344/DuCrl rats (Charles River) arrived at 7 weeks of age and were randomly distributed into three dosing groups (vehicle, 0.5 or 50 μg BPA/kg BW/d) and exposed to BPA via their drinking water, corresponding to approximately 0.5 or 50 μg/kg bw/day or vehicle control from gestational day 3.5 until weaning (postnatal day 21) using BPA (Sigma Aldrich, Missouri, USA, CAS 80-05-7) with ≥99% purity dissolved in ethanol (1% of final solution). The higher dose was chosen to match the tolerable daily intake set by the U.S. FDA (50 μg/kg/day), the lower dose approximately matches the highest estimated dietary intake of BPA in breast fed infants at the age of 0–6 month, which may reach 0.44–0.6 µg/kg bw/day, and the one of women of child bearing age, which can be up to 0.39 µg/kg bw/day^60^. Glass water bottles were used to minimize the background BPA exposure. Actual BPA intake and number of animals per treatment groups (previously published in Lejonklou, *et al*.^58^) is provided in Supplementary Table S4. The offspring were sacrificed at the age of 52 weeks, a time point chosen well after the dosing had been completed and the rats had reached sexual maturity, to investigate truly persistent changes after the chemical exposure had long ceased. The offspring were weighted at weaning and before sacrifice at 52 weeks of age (see Supplemental Table S1). Hippocampi were dissected from a total number of 56 rats, 28 males and 28 females with 8 animals in each BPA treatment and 12 in the control group and stored in RNA*later* RNA stabilization reagent (Qiagen, Hilden, Germany) to prevent RNA degradation. The study was carried out with the approval of the Uppsala ethical committee on Animal Research (ID # C26:13) and the guidelines laid down by the European Union Legislation were followed.

### Human data

The human data were collected from the Swedish Environmental Longitudinal, Mother and child, Asthma and allergy (SELMA) study, a pregnancy cohort study designed to investigate early life exposure to environmental chemicals and health outcomes related to growth, developmental and chronic diseases for the children. SELMA recruited pregnant women in the county of Värmland, Sweden between September 2007 and March 2010. Women who could read Swedish and were not planning to move out of the county were recruited at their first antenatal care visit; 8,394 pregnant women were identified, 6,658 were eligible and 2,582 (39%) agreed to participate. Detailed recruitment selection criteria and sample collection procedures have been published previously^34^. For the current study we used data from 318 randomly selected children at age 7 years, for which creatinine adjusted BPA levels had been measured in the 1^st^ trimester urine of their mothers, and oral swab samples and data on covariates had been collected for epigenetic analyses and statistical modelling, respectively. BPA levels were measured in its conjugated form in urine because urine levels are considerably higher than the ones in serum. This has two advantages: firstly, these measurements have been shown to be less sensitive to background contamination^61–63^ and secondly, it is more likely to highlight the between-individual variation.

First morning void urine samples were obtained from the 318 pregnant women in week 3–27 of pregnancy (median week 10, and 96% of the samples were taken before week 13) at enrolment to the study^34^. Urine samples were collected in supplied glass containers at home and transferred into polypropylene tubes without any other assisting equipment for easy transportation. Samples were stored at −20 °C before being processed at the laboratory at division of occupational and environmental medicine, Lund University, Sweden^64^. Quantitative analysis of urinary BPA concentrations was conducted using a triple quadrupole linear ion trap mass spectrometry (QTRAP 5500; AB Sciex, Foster City, CA, USA) coupled to a liquid chromatography system (UFLCXR, Shimadzu Corporation, Kyoto, Japan; LC/MS/MS). The samples were prepared according to the method presented in Bornehag, *et al*.^34^ and analysed according to a method described by Berge, *et al*.^65^. Urinary creatinine concentrations were analysed according to an enzymatic method described by Mazzachi, *et al*.^66^.

Blood samples were taken from the pregnant women at the time of enrolment and the serum aliquots were stored at −80 °C in a biobank. For cotinine levels, a biomarker for tobacco smoke exposure, prenatal serum samples were analysed for cotinine using LC-MS/MS as a biomarker for nicotine exposure, and used to assess smoking status. A detailed description of the method is presented in Lindh, *et al*.^67^. If cotinine levels were below 0.2 ng/mL, subjects were categorized as non-smokers; if cotinine levels were greater than 15 ng/mL, subjects were considered as active smokers; while in between (0.2–15 ng/mL), subjects were considered as passive smokers^68^.

Self-administered questionnaires were collected for background information including stressful events during pregnancy. We measured the maternal stress level using the items from one of our SELMA questionnaires. The participant was asked to answer if the mother had experienced at least one of the following situations during the pregnancy: (a) unemployment of the participant or the partner/spouse, (b) severe disease or injury in the participant, (c) severe disease or injury in the partner/spouse, children, sibling, or parents, (d) death of partner/spouse, children, sibling, or parents, (e) separation/divorce or any crisis in the relationship, and (f) financial crisis for the participant or the partner/spouse. Any situation at any time point during the pregnancy was coded as maternal stressful event.

Data on APGAR scores at the fifth minute for the child were imported from the Swedish National Birth Register.

The SELMA study is approved by Ethical Board of Uppsala, Sweden (2015-06-10, Dnr: 2015/177). Informed consents were obtained from all adult participants and guardians of under age participants. All human experimental methods and procedures were performed in accordance with the relevant ethical guidelines and regulations.

### Gene expression analysis

Extraction of total RNA from rat hippocampi was carried using the AllPrep® DNA/RNA/Protein Mini Kit (Qiagen, Hilden, Germany) in accordance with the manufacturer’s instructions. Quantity and quality of isolated RNA was assessed using a (TECAN, Grödig, Austria). Due to insufficient quality, 4 samples (1 male and 3 females) had to be excluded from the analysis. One μg of total RNA was treated with DNAseI (New England Biolabs, Ipswich, USA) and reverse transcribed using iScript™ Reverse Transcription Supermix (Bio-Rad Laboratories, Hercules, USA) according to manufacturer’s protocol. One μl of the resulting cDNA per reaction was used for real-time PCR using Rotor-Gene SYBR Green PCR kit on a Rotor-Gene 3000 (Qiagen, Hilden, Germany). Expression of *36B4* was used as reference gene and relative expression was calculated based on the delta-delta C_T_ method. Primers used for real-time PCR analyses are listed in Supplementary Table S5.

### DNA methylation analysis

Extraction of genomic DNA from rat hippocampi was carried out using the AllPrep® DNA/RNA/Protein Mini Kit (Qiagen, Hilden, Germany) in accordance with the manufacturer’s instructions. For human samples, isolation of genomic DNA from oral swabs was performed using BuccalAmp™ DNA extraction kit (Epicentre, Chicago, USA) according to manufacturer protocol. For 9 samples (4 males and 5 females), the DNA yield was insufficient for continued analyses. Bisulfite treatment was performed on 200 to 500 ng of extracted genomic DNA using the EZ DNA Methylation kit (Zymo Research, Irvine, USA). One μl of converted DNA was used for PCR amplification, and the PCR product was sequenced by pyrosequencing on a Pyromark Q24 (Qiagen, Hilden, Germany) in accordance with the manufacturer’s protocol, and the PyroMark Q24 software (Qiagen Pyromark Q24, version 5.0) was used for calculating percentage methylation at the analysed CpGs. To test the reliability of the assays, standard curves were performed using commercial genomic DNA standards (Qiagen, Hilden, Germany) (Supplementary Fig. S3). Primers used for pyrosequencing analyses are listed in Table S5.

### Statistical and bioinformatics analysis

For animal data, two-way ANOVA was used to compare level of gene expression between-groups and one-way ANOVA for comparing methylation levels within each group. The results were corrected using the Tukey’s post-hoc test. To adjust for possible batch effects or outliers in the DNA methylation measurements, we used rank-based inverse normal transformation to reprocess the raw data and re-analyze the effect of BPA exposure on methylation levels. We further used generalized linear model to regress methylation levels against both BPA exposure status and body weight change in order to control for growth rate. For correlation between DNA methylation and mRNA expression, Pearson analysis was used. The number of independent data points (n) is indicated in the figure legends of the corresponding graphs. Data is shown as mean + standard deviation (SD). The level of significance was selected at P < 0.05.

For human data, we implemented a multi-step statistical strategy to analyse the relationship between prenatal BPA exposure and methylations at *GRIN2B* at the age of 7 years. The steps included (1) clustering analysis to identify patterns of methylations, (2) ordered logistic regression to examine the effect of BPA exposure on methylations while adjusting for potential confounders, (3) stratified analysis by sex, and (4) structural equation models to perform multi-CpG-site analysis. The distributions of methylation levels at three CpG sites showed highly skewed-to-left distributions due to large numbers of samples showing 0% methylation. Such a skewed distribution of outcome might have caused the violation of normality assumption for residual errors in linear regression models, where methylation levels were regressed against prenatal BPA levels and other covariates. Therefore, the k-means clustering algorithm was used to identify clusters of methylation levels for each CpG site, as described in previous studies^69,70^. The appropriate number of clusters based on methylation levels at each CpG site was determined using the Calinski-Harabasz (CH) criteria (see Supplementary Fig. S3). The optimal value of clusters was chosen when CH pseudo-F value encountered the first abrupt increase. The drastic increase in this heuristic value is often used to indicate the identification of number of distinct clusters. Similar approaches have been described in other bioinformatics studies^71^.

The relationship between each cluster variable for each CpG site and prenatal BPA levels (expressed as a quartile variable) was then assessed using an ordered logistic regression model adjusted for potential covariates, including maternal smoking during pregnancy (based on cotinine concentration in prenatal serum), maternal self-reported stressful events during pregnancy (based on a retrospective questionnaire post partum), and APGAR scores (measured at birth at the fifth minute). Potential confounders/mediators were selected from pair-wise correlation tests (Spearman correlation test p-value < 0.05) or from previous evidence for their impacts on epigenetic patterns^72,73^. No covariate was found to remarkably impact the effect size of the key predictor, i.e. BPA levels (i.e., changing greater than 10% of the original regression coefficient).

Two-side alpha value = 0.05 was used to determine which CpG methylation levels could be predicted by the level of prenatal BPA. Finally, significant contributors were selected for methylation levels at each CpG site, and structural equation models (SEM) were used to examine the joint impact of predictors (with a focus on prenatal BPA levels) on multi-site methylation levels. The purpose of using SEM was to assess the relationship among multiple predictors and multiple outcomes simultaneously. The final SEM model was selected based on the Root Mean Square Error of Approximation (RMSEA) value with 0.1 as the cut-off. In addition to RMSEA, we also compared the selected model with the saturated model (i.e., as many estimated parameters as data points) to avoid over-fitting.

TF binding site prediction was performed using the JASPAR database^74^. Input sequence for rat was: GTGTGCACGCGCGT and for human: CGGGTGTGAGCGCGCGT. The list of all identified TF binding sites is provided as Supplementary Table S3.

### Data availability

The datasets generated from rats are available from the corresponding author on reasonable request. The De-identified SELMA exposure data may be available upon request in case a mutually agreed research plan and ethical approval documents are provided.

## Electronic supplementary material

Supplementary Material
