# Supplementary material for: Prenatal Bisphenol A Exposure is Linked to Epigenetic Changes in Glutamate Receptor Subunit Gene Grin2b in Female Rats and Humans
Source: Sci Rep. 2018 Jul 27;8:11315. doi: 10.1038/s41598-018-29732-9 (PMC6063959; doi:10.1038/s41598-018-29732-9)
Supplement: Supplementary file 1 — Supplementary Material [file 41598_2018_29732_MOESM1_ESM.pdf]

Supplementary Material:

Prenatal Bisphenol A Exposure is Linked to Epigenetic Changes in Glutamate Receptor Subunit Gene Grin2b in Female Rats and Humans

Ali Alavian-Ghavanini¶, Ping-I Lin¶, P Monica Lind, Sabina Risén Rimfors, Margareta H Lejonklou, Linda Dunder, Mandy Tang, Christian Lindh, Carl-Gustaf Bornehag, Joëlle Rüegg[1,2]*

¶ Authors contributed equally

* Corresponding author: Joëlle Rüegg, Swetox, Forskargatan 20, 151 36 Södertälje, Sweden. joelle.ruegg@swetox.se, +46(8)52488518

Table S1: Bodyweight and body length of one-year-old female and male Fischer 344 rat offspring that were included in both *Grin2b* expression and methylation analyses.

	Females				Males			
Outcome	Controls	0.5 µg BPA/kg/day	50 µg BPA/kg/day	ANOVA *p*-value	Controls	0.5 µg BPA/kg/day	50 µg BPA/kg/day	ANOVA *p*-value
Number of animals	12	8	8		12	8	8	
Weaning body weight (g)	38.7 ± 1.3	37.3 ± 1.0	37.1 ± 1.0	0.73$^{\#}$	41.0 ± 1.3	40.1 ± 0.6	38.9 ± 1.3	0.45
Final body weight (g)	218.3 ± 1.5	212.5 ± 2.2	223.0 ± 3.2	0.34$^{\#}$	455.0 ± 5.8	448.6 ± 6.4	447.8 ± 7.0	0.66
Body length (cm)	20.8 ± 0.1	20.7 ± 0.2	20.9 ± 0.2	0.69$^{\#}$	25.2 ± 0.1	25.2 ± 0.2	25.2 ± 0.2	0.99

Note: $^{\#}$Data not normally distributed; K-W *p*-value shown. All values are presented as mean ± SEM.

Table S2: Methylation and prenatal BPA levels in each cluster over three human CpG sites.

		All			Male			Female		
	Cluster	Number of subjects (N)	Mean methylation level (SD)*	Prenatal BPA level (95% CI)†	Number of subjects (N)	Mean methylation level (SD)*	Prenatal BPA level (95% CI)†	Number of subjects (N)	Mean methylation level (SD)*	Prenatal BPA level (95% CI)†
**hCpG1**	1st	175 (55.0%)	0 (0)	1.64 (1.45-1.82)	85 (51.5%)	0 (0)	1.62 (1.35-1.91)	90 (58.8%)	0 (0)	1.63 (1.41-1.88)
	2nd	62 (19.5%)	5.04 (0.62)	1.80 (1.40-2.31)	33 (20%)	5.05 (0.62)	2.04 (1.38-2.99)	29 (19.0%)	5.03 (0.64)	1.54 (1.12-2.11)
	3rd	54 (17.0%)	7.19 (0.87)	1.50 (1.12-2.01)	31 (18.8%)	7.21 (0.91)	1.26 (0.88-1.82)	23 (15.0%)	7.17 (0.84)	1.91 (1.15-3.18)
	4th	27 (8.5%)	11.67 (2.97)	1.76 (1.31-2.38)	16 (9.7%)	12.15 (3.67)	1.67 (1.12-2.49)	11 (7.2%)	10.97 (1.38)	1.90 (1.12-3.23)
**hCpG2**	1st	200 (62.9%)	0 (0)	1.63 (1.45-1.82)	97 (58.8%)	0 (0)	1.60 (1.34-1.91)	103 (67.3%)	0 (0)	1.66 (1.42-1.93)
	2nd	51 (16%)	5.09 (0.85)	1.74 (1.27-2.37)	31 (18.8%)	4.98 (0.95)	1.81 (1.15-2.83)	20 (13.1%)	5.28 (0.66)	1.63 (1.07-2.49)
	3rd	49 (15.4%)	8.08 (1.03)	1.57 (1.23-2.01)	27 (16.4%)	7.88 (0.95)	1.43 (1.02-2.00)	22 (14.4%)	8.33 (1.09)	1.78 (1.21-2.62)
	4th	18 (5.7%)	14.67 (3.89)	1.82 (1.34-2.47)	10 (6.1%)	14.22 (2.99)	1.98 (1.21-3.23)	8 (5.2%)	15.23 (4.96)	1.61 (1.05-2.48)
**hCpG3**	1st	263 (82.7%)	0 (0)	1.63 (1.46-1.81)	138 (83.6%)	0 (0)	1.59 (1.36-1.87)	125 (81.7%)	0 (0)	1.67 (1.45-1.92)
	2nd	14 (4.4%)	3.77 (0.38)	1.31 (0.81-2.13)	9 (5.5%)	3.82 (0.36)	1.45 (0.69-3.03)	5 (3.3 %)	3.67 (0.44)	1.10 (0.51-2.37)
	3rd	30 (9.4%)	5.21 (0.60)	1.94 (1.41-2.68)	14 (8.5%)	5.28 (0.73)	1.95 (1.22-3.12)	16 (10.5%)	5.17 (0.48)	1.94 (1.18-3.19)
	4th	11 (3.5%)	9.58 (1.86)	1.79 (1.17-2.74)	4 (2.4%)	8.82 (0.56)	2.16 (0.67-6.96)	7 (4.6%)	10.02 (2.24)	1.58 (0.93-2.71)

* The unit for methylation levels is %. SD indicates standard deviation.

† Geometric mean values are shown.

***Table S3**: Predicted transcription factor binding sites for the differentially methylated rat and human *Grin2b* promoter sequence, assessed using the JASPAR database.*

Matrix ID	Name	Score	Relative score	Sequence ID	Start	End	Strand	Predicted sequence
				**Rat**				
MA0259.1	ARNT::HIF1A	9.33022	0.944248677147	NC_005103.4:170000209-170000346	5	12	-	GCGCGTGC
MA0259.1	ARNT::HIF1A	5.60164	0.833016035713	NC_005103.4:170000209-170000346	5	12	+	GCACGCGC
MA0464.2	BHLHE40	5.93739	0.846536311237	NC_005103.4:170000209-170000346	4	13	+	TGCACGCGCG
MA0464.2	BHLHE40	5.75156	0.843449179455	NC_005103.4:170000209-170000346	4	13	-	CGCGCGTGCA
MA0823.1	HEY1	8.03575	0.859613562898	NC_005103.4:170000209-170000346	4	13	+	TGCACGCGCG
MA0823.1	HEY1	7.62576	0.852201792393	NC_005103.4:170000209-170000346	4	13	-	CGCGCGTGCA
MA0649.1	HEY2	6.56876	0.829149861227	NC_005103.4:170000209-170000346	4	13	+	TGCACGCGCG
MA0649.1	HEY2	6.21931	0.822543860312	NC_005103.4:170000209-170000346	4	13	-	CGCGCGTGCA
MA0738.1	HIC2	6.84208	0.880949089603	NC_005103.4:170000209-170000346	3	11	+	GTGCACGCG
MA1106.1	HIF1A	7.6051	0.871189403609	NC_005103.4:170000209-170000346	5	14	+	GCACGCGCGT
MA1106.1	HIF1A	5.61962	0.824439422835	NC_005103.4:170000209-170000346	3	12	-	GCGCGTGCAC
MA0058.3	MAX	5.27715	0.833442136573	NC_005103.4:170000209-170000346	4	13	-	CGCGCGTGCA
MA0058.3	MAX	4.7402	0.823640232202	NC_005103.4:170000209-170000346	4	13	+	TGCACGCGCG
MA0825.1	MNT	5.24008	0.815178540951	NC_005103.4:170000209-170000346	4	13	-	CGCGCGTGCA
MA0147.3	MYC	6.58738	0.805372883138	NC_005103.4:170000209-170000346	3	14	-	ACGCGCGTGCAC
MA0506.1	NRF1	9.0971	0.889334564697	NC_005103.4:170000209-170000346	2	12	-	GCGCGTGCACA
MA0506.1	NRF1	7.52342	0.869899942095	NC_005103.4:170000209-170000346	4	14	-	ACGCGCGTGCA
MA0506.1	NRF1	6.228	0.853901685919	NC_005103.4:170000209-170000346	3	13	+	GTGCACGCGCG
MA0831.1	TFE3	4.83888	0.817786937936	NC_005103.4:170000209-170000346	4	13	-	CGCGCGTGCA
MA0831.1	TFE3	3.91888	0.80033391606	NC_005103.4:170000209-170000346	4	13	+	TGCACGCGCG
				**Human**				
MA0471.1	E2F6	2.76015	0.801744622843	NC_018923.2:14099999-14100024	2	12	+	GGGTGTGAGCG
MA0162.2	EGR1	2.21576	0.803908917794	NC_018923.2:14099999-14100024	3	16	-	CGCGCGCTCACACC
MA0800.1	EOMES	7.17751	0.815982114252	NC_018923.2:14099999-14100024	1	13	+	CGGGTGTGAGCGC
MA0599.1	KLF5	1.71879	0.825118347309	NC_018923.2:14099999-14100024	1	10	-	CTCACACCCG

MA0498.2	MEIS1	3.07019	0.845762741388	NC_018923.2:14099999-14100024	4	10	-	CTCACAC
MA0801.1	MGA	12.3985	0.967704961012	NC_018923.2:14099999-14100024	2	9	+	GGGTGTGA
MA0506.1	NRF1	3.38873	0.818837328287	NC_018923.2:14099999-14100024	7	17	-	ACGCGCGCTCA
MA0014.3	PAX5	6.90319	0.8053899431	NC_018923.2:14099999-14100024	2	13	+	GGGTGTGAGCGC
MA0802.1	TBR1	7.98108	0.85327310593	NC_018923.2:14099999-14100024	2	11	+	GGGTGTGAGC
MA0805.1	TBX1	11.5212	0.938038345226	NC_018923.2:14099999-14100024	2	9	+	GGGTGTGA
MA0803.1	TBX15	11.3524	0.931336111457	NC_018923.2:14099999-14100024	2	9	+	GGGTGTGA
MA0688.1	TBX2	8.41702	0.853653201963	NC_018923.2:14099999-14100024	1	11	+	CGGGTGTGAGC
MA0690.1	TBX21	7.08414	0.829678540694	NC_018923.2:14099999-14100024	1	10	+	CGGGTGTGAG
MA0806.1	TBX4	10.6866	0.940194334718	NC_018923.2:14099999-14100024	2	9	+	GGGTGTGA
MA0807.1	TBX5	10.7467	0.945518371195	NC_018923.2:14099999-14100024	2	9	+	GGGTGTGA
MA0103.2	ZEB1	4.90603	0.828700312108	NC_018923.2:14099999-14100024	1	9	-	TCACACCCG

Table S4*:* Exposure details of BPA including the doses aimed for and actual doses that the dams consumed.

Exposure	[C] (n=13)	[0.5] (n=11)	[50] (n=9)
Dose in drinking water	0	0.0025 mg/L	0.25 mg/L
Dose aimed for	0	0.5 µg/kg bw/day	50 µg/kg bw/day
Actual dose (GD3.5-PND22)	0	0.404 µg/kg bw/day	40.1 µg/kg bw/day
Actual dose (GD3.5-birth)	0	0.272 µg/kg bw/day	26.9 µg/kg bw/day
Actual dose (birth-PND22)	0	0.530 µg/kg bw/day	52.7 µg/kg bw/day

Abbreviations: [C], Control; [0.5], 0.5 µg BPA/kg bodyweight/day; [50], 50 µg BPA/kg bodyweight/day; GD, Gestational day; PND, Postnatal day. Note that pups of one [0.5] dam was transferred to other [0.5] dams at PND4.

Table S5: Sequences of the oligonucleotides used for this study.

**Methylation analysis**		
rGRIN2B-For<br>*rGRIN2B*-Rev	5´- ATAGGAGGAGAAATTTAAGAGGTAG - 3´<br>5´- TAACCACTATCTTACTCCCCTCAC - 3´	PCR amplification
hGRIN2B-For<br>*hGRIN2B*-Rev	5´- TGATTTAGGGGGGAGGAGAAATT - 3´<br>5´- AAACTACCTCCCCCAAAATCTTAACA - 3´	PCR amplification
rGRIN2B (CpG1-3)<br>*rGRIN2B (CpG5-7)*<br>*hGRIN2B*	5´- GAGGTAGGGTGTGTGTGTA - 3´<br>5´- GAAAAGTTTAATTTGAGTT- 3´<br>5´- GGAAGATATTGTTTTTGTTTTTAG - 3´	Pyrosequencing
**Gene expression analyses**		
rGRIN2B-For<br>*rGRIN2B*-Rev	5’ - TGAAGATGGCTACCAGATGC - 3’<br>5’ - GCAGGGACTTGTCCTTCCAT - 3’	
R36B4-For<br>*R36B4-Rev*	5´- TTCCCACTGGCTGAAAAGGT - 3´<br>5´- GCCGCAGCCGCAAATGC - 3´	

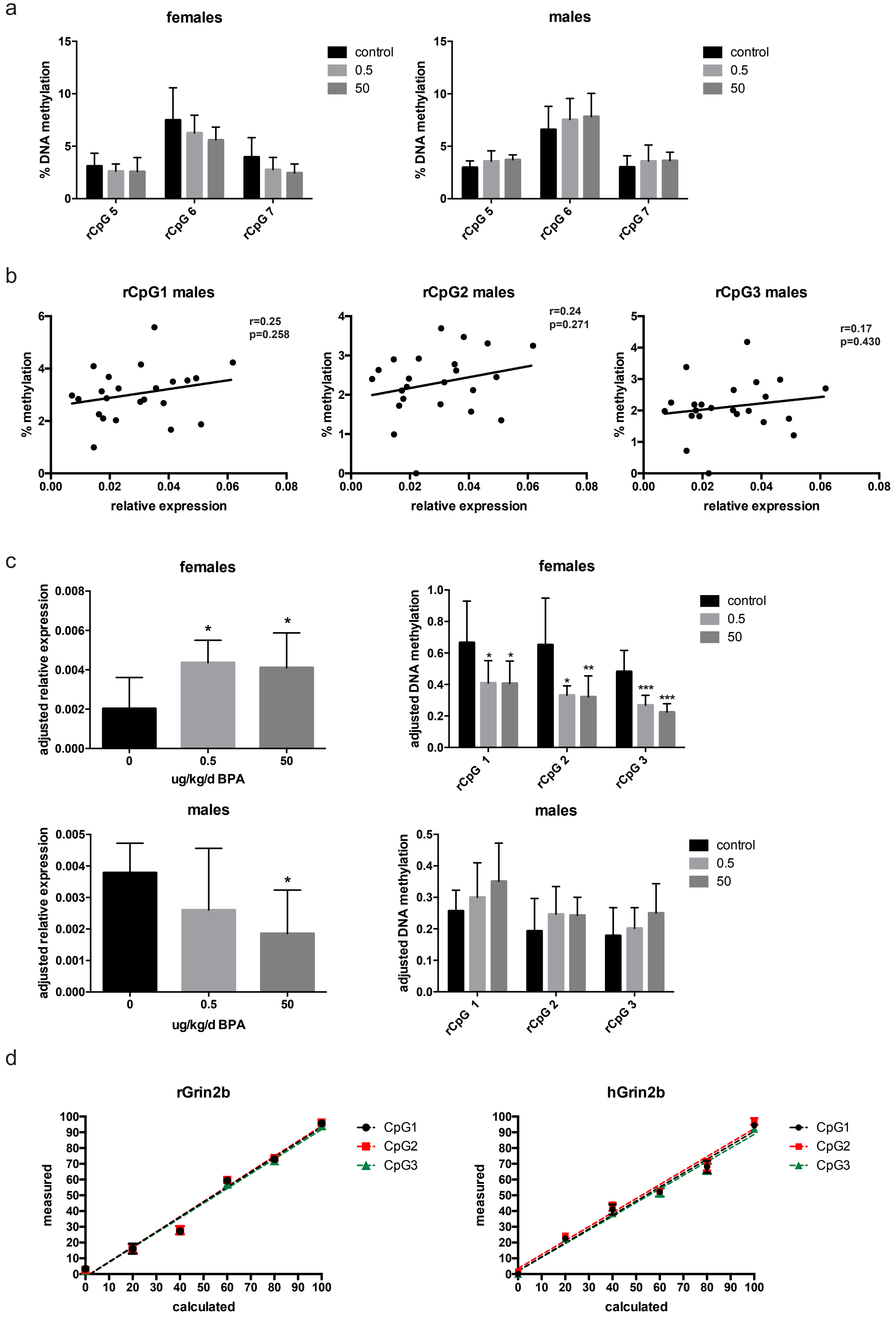
a
females
males
% DNA methylation
control
0.5
50
rCpG 5
rCpG 6
rCpG 7
b
rCpG1 males
rCpG2 males
rCpG3 males
r=0.25
p=0.258
r=0.24
p=0.271
r=0.17
p=0.430
% methylation
relative expression
c
females
males
adjusted relative expression
ug/kg/d BPA
adjusted DNA methylation
control
0.5
50
rCpG 1
rCpG 2
rCpG 3
d
rGrin2b
hGrin2b
measured
calculated
CpG1
CpG2
CpG3

Figure S1: **a**) DNA methylation analysis of rCpGs 5-7 in the promoter region of *Grin2b* assessed by bisulfite pyrosequencing in hippocampus of 52 weeks old rats developmentally exposed to 0.5 or 50 μg/kg bw/day BPA or vehicle control. Bars show mean and standard deviation of % methylation for female (control: n=8, 0.5: n=7, 50: n=8) and male (control: n=8, 0.5: n=8, 50: n=8) animals. **b**) Correlation between relative Grin2b expression and methylation CpG 2 and 3 in female and CpG 1, 2, and 3 in male rat hippocampus. **c**) Relative *Grin2b* expression (left panels) and % DNA methylation (right panels) adjusted for growth rate using a stratified approach whereby for each individual, expression or methylation values were divided by the growth rate and subsequently analysed using one-way ANOVA. **d**) Standard curves for the Pyrosequencing assays of rat and human *Grin2b*. $^{*}p < 0.05$, $^{**}p < 0.01$, $^{***}p < 0.001$ treatment animals compared to controls.

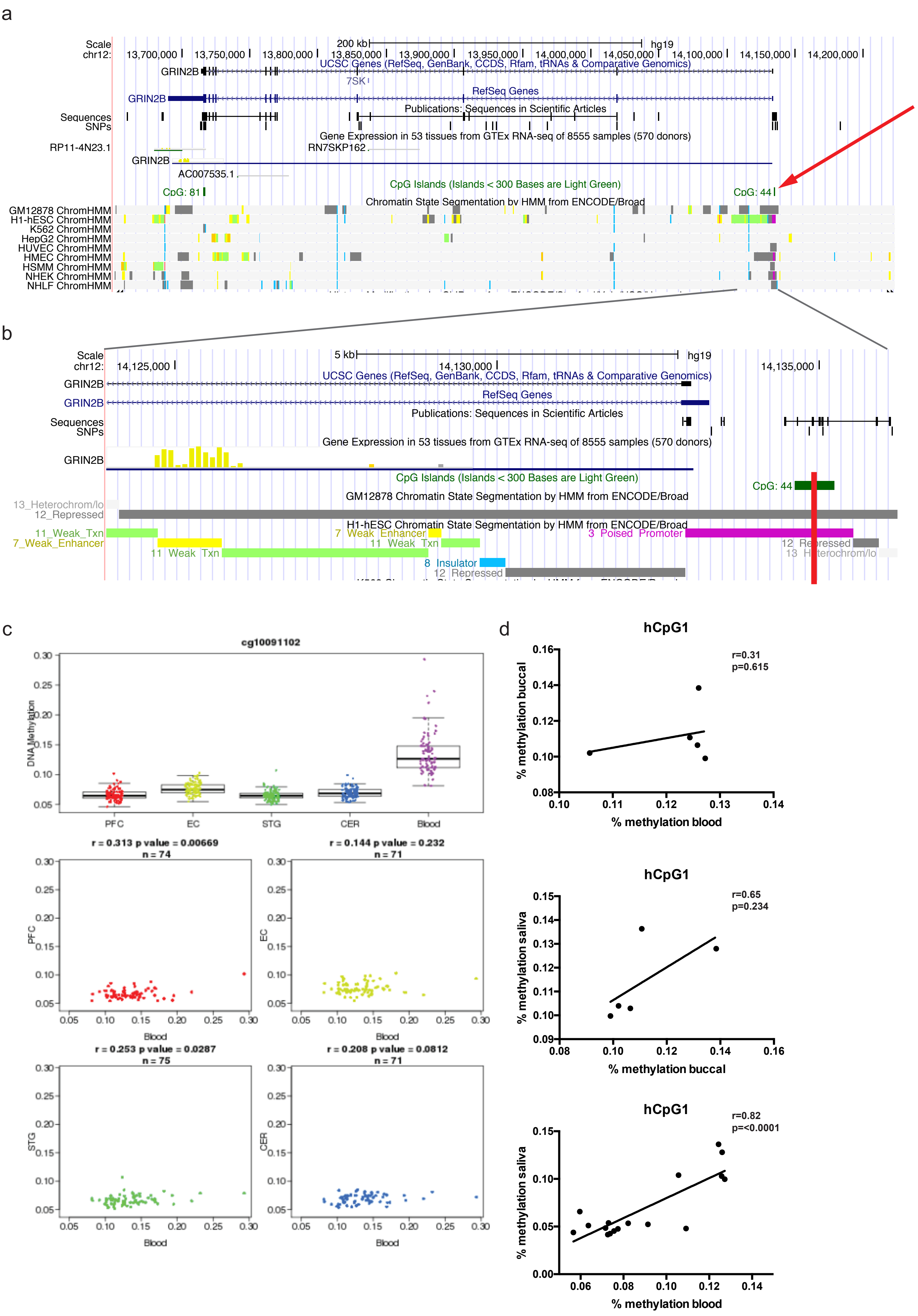
a
Scale
chr12:
200 kb
hg19
13,700,000
13,750,000
13,800,000
13,850,000
13,900,000
13,950,000
14,000,000
14,050,000
14,100,000
14,150,000
14,200,000
UCSC Genes (RefSeq, GenBank, CCDS, Rfam, tRNAs & Comparative Genomics)
GRIN2B
7SK
RefSeq Genes
Publications: Sequences in Scientific Articles
Sequences
SNPs
Gene Expression in 53 tissues from GTEx RNA-seq of 8555 samples (570 donors)
RP11-4N23.1
RN7SKP162
AC007535.1
CpG Islands (Islands < 300 Bases are Light Green)
CpG: 81
CpG: 44
Chromatin State Segmentation by HMM from ENCODE/Broad
GM12878 ChromHMM
H1-hESC ChromHMM
K562 ChromHMM
HepG2 ChromHMM
HUVEC ChromHMM
HMEC ChromHMM
HSMM ChromHMM
NHEK ChromHMM
NHLF ChromHMM
b
Scale
chr12:
5 kb
hg19
14,125,000
14,130,000
14,135,000
GM12878 Chromatin State Segmentation by HMM from ENCODE/Broad
13_Heterochrom/lo
12_Repressed
H1-hESC Chromatin State Segmentation by HMM from ENCODE/Broad
11_Weak_Txn
7_Weak_Enhancer
3_Poised_Promoter
8_Insulator
c
cg10091102
DNA Methylation
PFC
EC
STG
CER
Blood
r = 0.313 p value = 0.00669
n = 74
r = 0.144 p value = 0.232
n = 71
r = 0.253 p value = 0.0287
n = 75
r = 0.208 p value = 0.0812
n = 71
d
hCpG1
r=0.31
p=0.615
% methylation buccal
% methylation blood
hCpG1
r=0.65
p=0.234
% methylation saliva
% methylation buccal
hCpG1
r=0.82
p=<0.0001
% methylation saliva
% methylation blood

Figure S2: **a)** Genome Browser[1] graphic showing the genomic context of the human *GRIN2B* gene. Two CpG islands were found, one containing the analysed region (red arrow) and one in the last exon. In the lower part, predicted regulatory elements are shown based on relevant information assessed in the ENCODE project. The only predicted promoter region (pink) contains the analysed region. **b)** Enlarged representation of the region around the *GRIN2B* transcriptional start site including the CpG island, the predicted promoter region (pink), and the analyzed sequence (red bar). **c)** Plot extracted from the Blood Brain DNA Methylation Comparison Tool [2] showing *GRIN2B* methylation at hCpG 1 (Illumina 450K probe cg 10091102) in blood (purple dots), prefrontal cortex (PFC, red dots), entorhinal cortex (EC, yellow dots), superior temporal gyrus (STG, green dots), and cerebellum (CER, blue dots) assessed in matched samples (n=75) using the Illumina 450K platform [3], as well as correlations between DNA methylation at hCpG 1 in blood and the 4 different brain region. **d)** Correlation between *Grin2b* methylation at hCpG 1 in DNA isolated from blood and buccal swabs (top panel), buccal swabs and saliva (middle panel), or saliva and blood (lower panel). Values were extracted from published data sets including matched samples for hCpG 1 methylation assessed using the Illumina 450K platform: [4] providing data for saliva-blood comparison (n=12) and providing data for saliva-blood, buccal-blood, and saliva-buccal comparison (all n=5).

Supplementary Figure S3

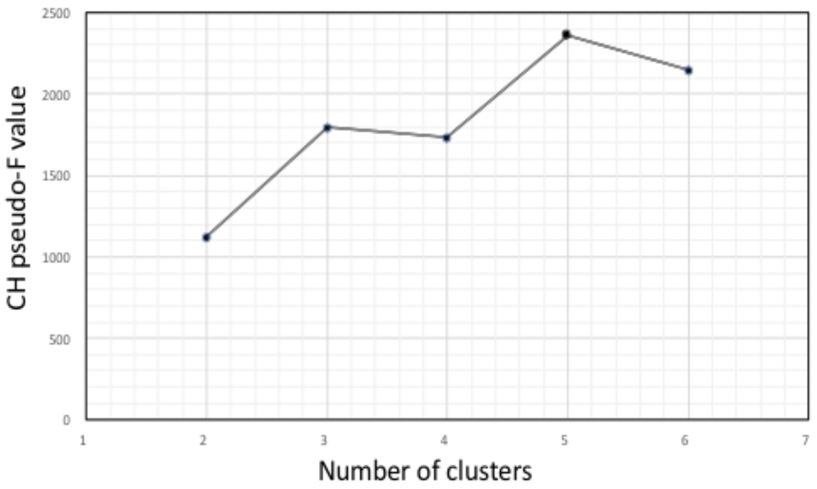

Figure S3. This figure shows how we determined the number of cluster of methylations levels at first CpG site, using the Calinski-Harabasz (CH) criteria. The sudden change in the CH value often indicates the optimal number of clusters.

References:

1 Karolchik, D. *et al.* The UCSC Genome Browser database: 2014 update. *Nucleic acids research* **42**, D764-770, doi:10.1093/nar/gkt1168 (2014).

2 Eilis Hannon, K. L., Leonard Schalkwyk, and Jonathan Mill. *Blood Brain DNA Methylation Comparison Tool*, <http://epigenetics.iop.kcl.ac.uk/bloodbrain/> (2017).

3 Hannon, E., Lunnon, K., Schalkwyk, L. & Mill, J. Interindividual methylomic variation across blood, cortex, and cerebellum: implications for epigenetic studies of neurological and neuropsychiatric phenotypes. *Epigenetics : official journal of the DNA Methylation Society* **10**, 1024-1032, doi:10.1080/15592294.2015.1100786 (2015).

4 Langie, S. A. *et al.* Whole-Genome Saliva and Blood DNA Methylation Profiling in Individuals with a Respiratory Allergy. *PLoS One* **11**, e0151109, doi:10.1371/journal.pone.0151109 (2016).
